# Internal morphology of 101 mandibular canines of a Swiss-German population by means of micro-CT: An ex vivo study

**DOI:** 10.1038/s41598-021-00758-w

**Published:** 2021-10-28

**Authors:** Thomas Gerhard Wolf, Andrea Lisa Anderegg, David Haberthür, Oleksiy-Zakhar Khoma, Sven Schumann, Nane Boemke, Richard Johannes Wierichs, Ruslan Hlushchuk

**Affiliations:** 1 Department of Restorative, Preventive and Pediatric Dentistry, School of Dental Medicine, University of Bern, Freiburgstrasse 7, 3010 Bern, Switzerland; 2 Department of Periodontology and Operative Dentistry, University Medical Center of the Johannes Gutenberg-University Mainz, Mainz, Germany; 3 Institute of Anatomy, University of Bern, Bern, Switzerland; 4 Institute for Microscopic Anatomy and Neurobiology, University Medical Center, Johannes Gutenberg-University, Mainz, Germany

**Keywords:** Dental pulp, Root canal treatment, Root canal treatment, Dental clinical teaching, Extended skills training in dentistry

## Abstract

The aim of this study was to investigate the root canal system morphology by means of a root canal configuration (RCC) classification described with a four-digit system, the physiological foramen geometry and accessory canal frequency and morphology, of 101 mandibular canines (MaCa) of a Swiss-German population by means of micro-computed tomography. Micro-CT examination of the MaCa was performed and the obtained images analyzed with a 3D imaging software. In single-rooted MaCas, the most frequently observed RCCs were 1-1-1/1 (74.5%) and 1-1-1/2 (14.3%). Seven other RCCs were less frequently observed with a frequency from 4.1 to 1.0%. One physiological foramen was observed in 80.6% of the MaCas, two in 16.3%, three in 1.0% and four in 2.0%. Accessory and connecting canals were apparent only in the middle and apical root thirds. Two-rooted MaCas occurred less frequently (n = 3). When one physiological foramen was present, the mean size of the narrow and wide diameters were 0.28 mm (± 0.07) and 0.40 mm (± 0.11), while the distance between physiological and anatomical foramen was 0.45 mm (± 0.17). MaCas are predominantly single-rooted teeth with a 1-1-1/1 or 1-1-1/2 RCC. Most MaCas had one physiological foramen with an oval shape.

## Introduction

Mühlreiter^1^ first described the tooth morphology by means of sectioning in axial planes. While investigative methods have considerably improved since, the interest in and importance of the root canal system morphology has not diminished. It is essential for operators to be familiar with the root canal system morphology and to be able to recognize without delay any possible variation of it in order to minimize endodontic treatment failure^2,3^. Moreover, understanding that the morphology complexity of the root canal system may be substantially masked by the relatively simple and uniform radiological anatomy of the external root surface^4^ reports a meaningful clinical benefit. Various root canal system morphology research methods as radiographic analysis, tooth clearing, microscopy, and macroscopic sectioning^5–7^ have been implemented. The two most recently introduced research methods are cone beam computed tomography (CBCT) and micro-computed tomography (micro-CT). CBCT has been used to examine in vivo the root canal system morphology of mandibular canines (MaCa)^8,9^ and micro-CT has been used to investigate different dental root canal systems and foramina morphologies^10–16^. However, micro-CT has proven, in combination with a 3D imaging rendering software, that it is a reproducible, non-destructive and non-invasive research method, thus, it has been reported to be the gold standard research method for this purpose^17,18^. Furthermore, micro-CT, due to its high resolution, enhances a precise recognition of the root canal configuration (RCC), thus, the use of a more comprehensive RCC description method^10^ than the ones frequently used reported by Vertucci^19^ and Weine et al.^3^. The root canal configuration of MaCas has not, as far as we are aware of, been investigated by means of the four-digit classification system and micro-CT; hence, the aim of this investigation was to investigate the RCC, physiological foramen anatomy and accessory and connecting canals number of MaCas in a Swiss-German population by means of micro-CT.

## Materials and methods

### Tooth selection

One hundred and one extracted human permanent mandibular canines (MaCa) were collected from university medical centers and private dental offices in Switzerland and Germany. All teeth were extracted for reasons unrelated to this investigation. All teeth investigated in this study were declared as “excess material”, thus, they could be used for scientific purposes without requiring any additional approval of the corresponding ethics committee (Contract General Terms [AVB], §14 Organ explantation/further use of body material, Status: 1 April, 2017). The teeth investigated were selected by two independent observers (A.L.A., T.G.W.). The inclusion criteria were teeth that could be clearly identified, according to their coronal morphological dimensions^20^, as MaCa, had completely developed roots and lack of coronal or radicular resorption, caries, root fracture and previous endodontic treatment were identified. Any adherent soft or hard tissue and calculus were removed with ultrasonic (Pieton 150; EMS Dental, Nyon, Switzerland) and manual scalers. The teeth were then stored in a 2% chloramine solution (Sigma-Aldrich, St. Louis, MI, USA) until the micro-CT examination was performed.

### Micro-computed tomography investigation

The 101 MaCas were imaged with the automated sample changer on a Bruker SkyScan 1272 high-resolution micro-tomography device and integrated software (Bruker microCT, Control software version 1.1.19, Kontich, Belgium). The device is equipped with a Hamamatsu L11871-20, X-ray source (Hamamatsu Photonics, Hamamatsu; Shizuoka, Japan) and a Ximea xiRAY16 camera (XIMEA GmbH; Münster, Germany). The X-ray source was set at 80 kV and 125 µA and the spectrum was filtered by 1 mm of aluminum. A set of five stacked scans overlapping the height of each sample was recorded. Each stack was recorded with 482 projections (TIFF, 1632 × 1092 pixels) every 0.4° over a 180° sample rotation. Three projections, exposed for 950 ms, were averaged in order to reduce image noise. A stack was scanned in approximately 40 min, resulting in a final data set with of an isometric voxel size of 10.0 µm^11^. The projection images were then subsequently reconstructed into a 3D stack (NRecon 1.7.4.6; Bruker microCT, Kontich Belgium) and analyzed with the Jupyter notebook tool^21^. The automated segmentation and root canal system morphology description preparation method of the tooth datasets has been previously described^11^. The middle slice of the axial, frontal and sagittal planes was extracted and the maximum intensity projections (MIP) of each plane were generated for rapid visual assessment of each tooth. Based on the extent of the MIP, the datasets were cropped to their minimal size to facilitate further processing. The apical 3.5 mm of the MaCas were examined by re-slicing this smaller area into sagittal slices. The dentin-enamel junction was automatically detected and the lower part of each tooth was split into four equidistant slices from the most apical region to the enamel-cement junction^11^.

### Root canal configuration method

A four-digit root canal system configuration (RCC) classification was used^10^, in which the roots are divided into thirds. The first, second and third digits denote the coronal, middle and apical thirds, respectively. Each digit represents the root canal number at the coronal limit of the corresponding third. The fourth digit is separated with a slash (/) and stands for the number of physiological foramina. The apical area was investigated as previously described^16^. A physiological foramen is defined as the one that originates from a main root canal and has a diameter of ≥ 0.2 mm^10^. All apical foramina with a diameter < 0.2 mm were classified as accessory foramina. The physiological as well as the accessory foramina were analyzed in the axial and sagittal planes. The anatomical (widest diameter) and physiological (narrowest diameter) foramina were determined and the distance between both was measured^10,16^ with a 3D imaging software Fiji^22^. The physiological foramen shape was defined as oval when the difference between narrow and wide diameters was ≥ 0.02 mm^23^. The number of connecting and accessory canals was determined and classified according to their location in either the coronal, middle or apical root thirds. A connecting canal was defined as the one that connects a root canal with the same or another root canal without emerging into the periapical tissue. The results of this study are descriptively expressed with absolute and relative values.

## Results

### Micro-CT investigation

**Figure 1:**
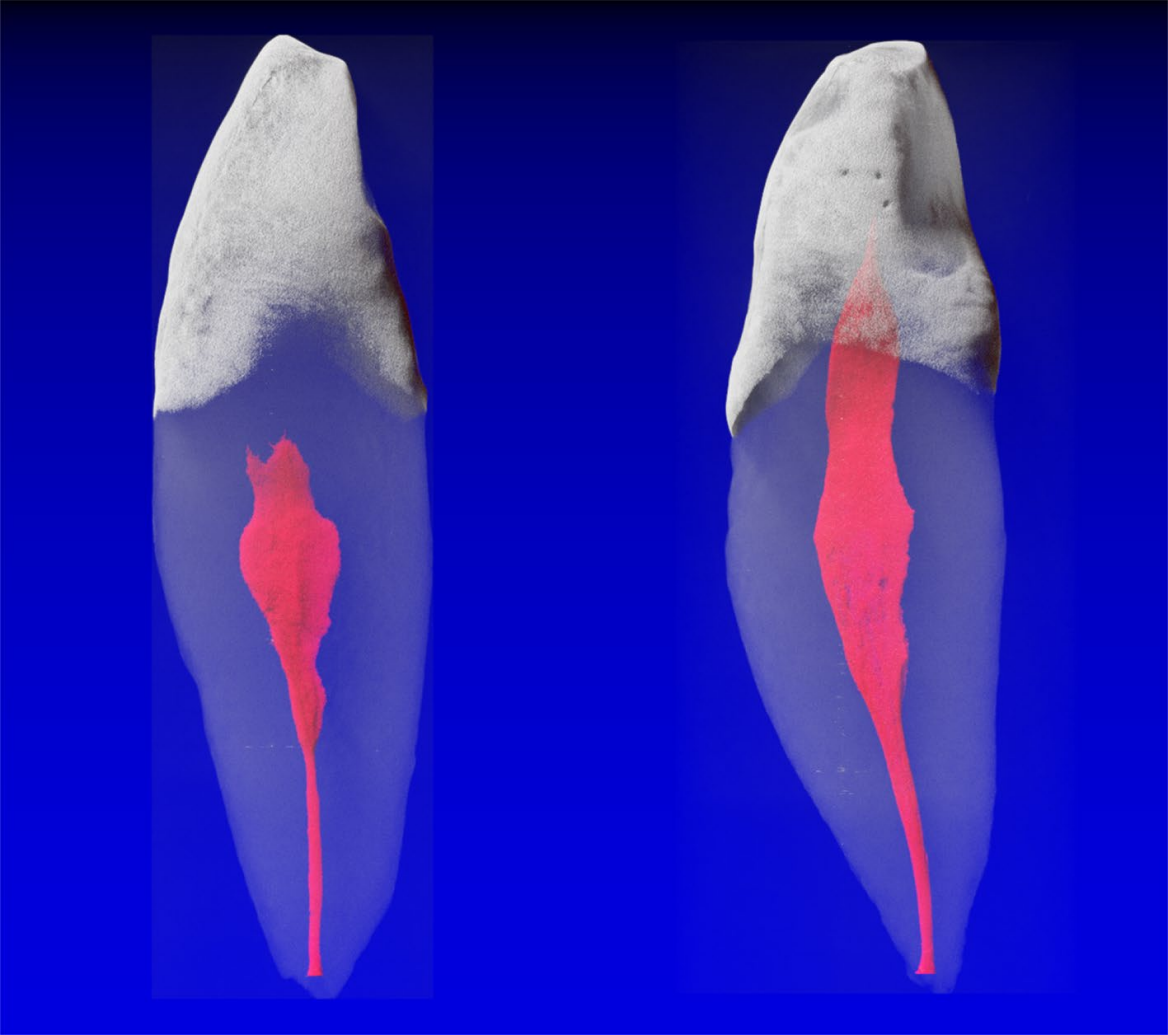
Mandibular canines with a 1-1-1/1 root canal configuration. The root canals observed were most of the time wide (in a mesio-distal projection); however, the tended to narrow as they reached the apical third.

**Figure 2:**
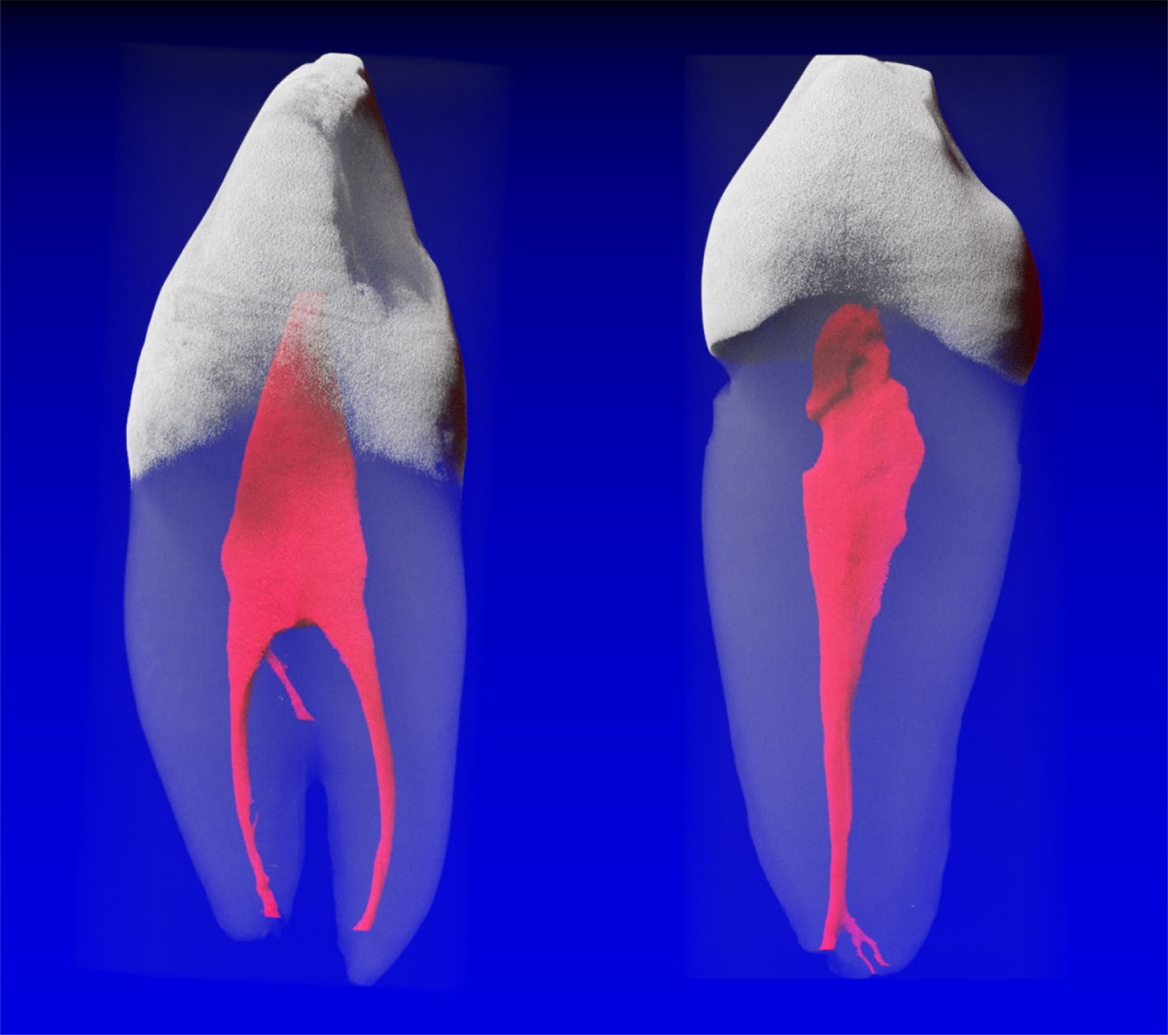
Mandibular canine with a 1-2-2/2 (left) root canal configuration (RCC) and one accessory (interradicular) canal in the middle third. A 1-1-1/1 RCC (right) with one accessory canal which divides into two accessory canals/foramina can be observed.

The results of 101 mandibular canines (MaCa) showed 98 single-rooted and three two-rooted teeth. The roots of the two-rooted MaCas divided into two separate roots in the middle or apical third; therefore, they were considered as two-rooted MaCa in this study. The 3D reconstructions of the micro-CT scans allowed a visualization of the enamel in white, the pulp space in red and the dentin in transparent gray (Figs. 1, 2). The root canal configurations (RCCs) results of the single and two-rooted MaCas are shown in Table 1. The most frequently RCCs observed in MaCas were the 1-1-1/1 (74.5%) one (Fig. 1) followed by the 1-1-1/2 (14.3%), 1-2-1/1 (4.1%) and 1-1-1/4 (2.1%) RCCs. Five other RCCs were less frequently (1.0%) observed (Table 1). Of the two-rooted MaCa, 66% showed a 1-1-1/1 configuration in the buccal and lingual roots (Fig. 2). RCCs 1-2-1/1 and 1-1-1/2 were observed once (33.3%) in the buccal and lingual roots, respectively, of two rooted MaCas (Table 1).

**Table 1:** Root canal configuration (RCC) of 101 mandibular canines.

Root(s)	RCC	Frequency	
		Absolute	Mean
Single-rooted (n = 98)	1-1-1/1	73	74.5
	1-1-1/2	14	14.3
	1-1-1/3	1	1.0
	1-1-1/4	2	2.1
	1-1-2/1	1	1.0
	1-2-1/1	4	4.1
	1-2-1/2	1	1.0
	1-2-2/2	1	1.0
	2-3-1/1	1	1.0
Two-rooted (n = 3)			
Buccal	1-1-1/1	2	66.6
	1-2-1/1	1	33.3
Lingual	1-1-1/1	2	66.6
	1-1-1/2	1	33.3

The RCC digits from left to right describe the root canal number from the coronal, middle to the apical third of the root. The fourth digit, separated with a slash (/), depicts the physiological foramina number (n/total = 101, n/single-rooted = 98, n/two-rooted = 3).

Most single rooted MaCas had one physiological foramen (80.6%) and four to one accessory canals (15.3–1.0%). The buccal root of two-rooted MaCas had one (100%) and the lingual one (66.6%) and two (33.3%) physiological foramina. Only one accessory canal was observed in the buccal root (33.3%) of two-rooted MaCas, otherwise no accessory canals could be observed (Table 2). The frequency of accessory and connecting canals in MaCas in this investigation was unusual (Table 3). In single rooted MaCas one accessory canal was identified in 13 MaCas (12.8%); six (5.9%) and seven (6.9%) in the apical and middle thirds, respectively. Only two connecting canals (2.0%) were observed, all of them located in the middle third. One loop-type connecting canal (emerging from and returning to the same root canal) and one communicating canal (communicating two different root canals in the same root) were observed (Table 3). The physiological foramina diameter sizes of single-rooted mandibular canines and the distance between the anatomical apex and the physiological foramen is shown in Table 4. Recommendations for the final preparation of the physiological foramina size (MAF), based on the diameter size results obtained, are suggested (Table 4). The most frequently observed physiological foramina shape in single-rooted MaCas with one foramen was an oval shape (91.1%) followed by the round (7.6%) and irregular (1.3%) shapes. When more than one physiological foramen was observed, all foramina had an oval shape (Table 5).

**Table 2:** Absolute (n) and mean (%) frequency of the physiological and accessory foramina frequency observed under micro-CT in mandibular canines (n = 101; n/one root = 98, n/two roots = 3; F = foramina frequency, 1R = single-rooted, 2R = two-rooted, B = buccal root, L = lingual root, Ph = physiological foramina, Ac = accessory canals).

Physiological and accessory foramina frequency												
F	1R-Ph		1R-Ac		2R-Ph/B		2R-Ph/L		2R-Ac/B		2R-Ac/L	
	n	%	n	%	n	%	n	%	n	%	n	%
0	–	–	66	67.3	–	–	–	–	2	66.6	3	100.0
1	79	80.6	15	15.3	3	100.0	2	66.6	–	–	–	–
2	16	16.3	8	8.2	–	–	1	33.3	1	33.3	–	–
3	1	1.0	8	8.2	–	–	–	–	–	–	–	–
4	2	2.0	1	1.0	–	–	–	–	–	–	–	–

**Table 3:** Absolute and mean frequency of connecting and accessory canals in the coronal, middle and apical root thirds of single-rooted (n = 98) and two-rooted (n = 3) mandibular canines (n = 101).

Single rooted	n	%	
**Accessory canals**			
None	88	87.1	
Coronal	0	0.0	
Middle	7	6.9	
Apical	6	5.9	
**Connecting canals**			
None	96	98.0	
1-1-1/1	0	0.0	
1-1-1/2	0	0.0	
1-1-1/3	0	0.0	
1-1-1/4	0	0.0	
1-1-2/1	0	0.0	
1-2-1/1	1	1.0	(Type L)
1-2-1/2	0	0.0	
1-2-2/2	0	0.0	
2-3-1/1	1	1.0	(Type C)
1-1-1/1	0	0.0	
1-2-1/1	0	0.0	
1-1-1/1	0	0.0	
1-1-1/2	0	0.0	
**Two-rooted**			
**Accessory canals**			
None	2	2.0	
Coronal	0	0.0	
Middle	1	1.0	
Apical	0	0.0	
**Connecting canals**			
None	1	1.0	
1-1-1/1	1	1.0	
1-1-1/2	1	1.0	
1-2-1/2	0	0.0

**Table 4:** Statistical description of the physiological foramen diameter dimensions (mm) and distances between the anatomical and physiological foramen in single-rooted mandibular canines (n = 98) and final preparation size (master apical file [MAF]; ISO instrument tip diameter) recommendations (W = wide diameter; N = narrow diameter, D = Distance between physiological foramen and anatomical foramen; SD = standard deviation).

Foramina (n)	1			2			3			4		
Diameter/Distance (mm)	W	N	D	W	N	D	W	N	D	W	N	D
Ø	0.40	0.28	0.45	0.38	0.22	0.16	0.32	0.17	0.17	0.32	0.18	0.18
SD	0.11	0.07	0.17	0.11	0.07	0.05	0.06	0.02	0.02	0.09	0.03	0.04
Max	0.88	0.63	0.94	0.74	0.40	0.29	0.38	0.21	0.19	0.49	0.24	0.24
Min	0.21	0.14	0.10	0.22	0.12	0.10	0.25	0.15	0.14	0.23	0.13	0.13
Total (n)	79			16			1			2		
MAF	45/50			40/45			40			40

**Table 5:** Absolute and relative physiological foramina shape frequency of single-rooted mandibular canines (n = 98) according to the foramina number.

Foramina (n)/Shape	1		2		3		4	
	n	%	n	%	n	%	n	%
Oval	72	91.1	16	100.0	1	100.0	2	100.0
Round	6	7.6	0	0.0	0	0.0	0	0.0
Irregular	1	1.3	0	0.0	0	0.0	0	0.0
n	79		16		1		2	

## Discussion

The aim of this study was to examine the root canal system morphology of mandibular canines (MaCa) with a solid sample size (n = 101), thus, allowing a reliable statistical evaluation. Different research methods, such as decalcifying and ink dye^7^, radiographic imaging^5^, cross-sectional^24^, CBCT imaging^8,9,25–28^ and micro-CT imaging^13,14^, have been used to examine root canal morphologies. The cross-sectional and decalcification methods demand, at least to a certain extent, a sample destruction; furthermore, with the cross-sectional method, due to the slice thickness, an accurate reconstruction of the internal morphology becomes burdensome. The radiographic imaging method is, due to its intrinsic low resolution, a difficult to interpret and reproduce, thus, a rather subjective method. Therefore, it is not surprising that given the progress of three-dimensional imaging methods, methods such as cross-sectioning, decalcification and conventional two-dimensional radiographs are being replaced through minute, and hence, objective root canal morphology research methods^18^. Recent studies considering the morphology of MaCas, investigating a relatively large sample size, have been in vivo performed by means of CBCT imaging^8,9,25–28^. CBCT examinations seem to be an accurate in vivo method for analyzing the root canal morphology; however, CBCT does not provide images with a high-resolution as those of micro-CT^10,15,18^. It is frequently accepted that actually the most accurate ex vivo method to investigate root canal configurations (RCC) is the micro-CT analysis supported with a 3D reconstruction, as it is a non-destructive, non-invasive, and reproducible method^17,18^. Micro-computed tomography is able to provide accurate and quantifiable results, thus, is considered as the gold standard when investigating RCCs^18^. However, relative few studies have investigated the MaCa root canal morphology by means of micro-computed tomographic imaging^12–14,29^. The RCC description systems proposed by Vertucci^19^ and Weine et al.^3^ have been widely reported in the literature. However, different computer-assisted imaging techniques, such as micro-CT, have enhanced the possibility to depict minute RCCs details that can hardly be accurately classified with the afore mentioned RCC systems. This investigation takes advantage of an accurate four-digit RCC system based on the root sectioning into thirds and the use of a fourth digit to describe the physiological foramina number identified^10^. The 1-1-1/1 RCC (74.5%) frequency obtained in this investigation in MaCas is in agreement with the ones of previous reports^9,25^. However, this RCC has been also reported with a relative lower frequency (35.8%) in female patients^26^. These differences could be explained by the different research methodologies and populations investigated. The second most frequently RCC observed in this study was the 1-2-1/2 (14.3%), which describes a root canal that splits into two, merges apically; yet, ends with two physiological foramina. This RCC has been seldom reported by other authors^27,28^. In our opinion, the lack of these findings, supports the fact that minute RCC details can be only/easier identified with micro-CT when compared with other research methodologies.

In 67.3% of the investigated MaCas, no accessory foramina were found. One accessory foramen was observed in 15.3%, two and three in 8.3%, and four accessory foramina were identified in one MaCa. Most MaCas examined had neither accessory (89.1%) nor connecting (96.0%) canals. The information reported in the literature, when considering the frequency of accessory foramina and accessory and connecting canals in MaCas, is scarce; yet, the findings obtained in this investigation are in agreement to some extent with the ones of a previous report^14^. The overall mean distance between the anatomical and physiological foramina of MaCas in this research was of 0.24 mm (± 0.07) and a maxima and minima of 0.94 and 0.10 mm, respectively. Higher results (Ø 0.95 mm; ± 0.50; max = 2.38 and min = 0.13 mm) when compared with this study have been reported^30^. These differences could be explained through the investigation methodology differences. Although further distance information between the anatomical and physiological foramina of MaCas is scarce, it is important for the operator to be aware that in both investigations, the ranges between the minima and maxima as well as the standard deviations are high. Thus, this regarding, it is not advisable to make a radiological clinical recommendation for the clinician. The most frequently physiological foramina shape observed in this investigation, alike another prior report on mandibular first premolars^31^, was the oval one (91.1–100%). Direct comparison between these results should be cautiously exercised due to the different tooth types investigated^31^ and to the fact that different research groups^32,33^ do no make any tooth type distinction. To the best of our knowledge, there have been few attempts^15,16,23^ to report a clear definition of the foramina shape. In this investigation, an oval foramina shape was defined as the one in which the difference between the wide and narrow diameters was of ≥ 0.02 mm; otherwise, for the sake of the inherent morphology of teeth, it was defined as a round shape. An irregular shape was the one in which more than two different diameters were encountered.

A single physiological foramen was the most frequently observed one in this research (80.6%), followed by two (16.3%); yet, three physiological foramina could only be observed in one tooth and four foramina in two teeth. When one physiological foramen was observed, the wide and narrow average diameters were 0.40 and 0.28 mm, respectively. A small diameter mean reduction was observed in MaCas with two (0.38 and 0.22 mm), three (0.32 and 0.17 mm) and four (0.32 and 0.18 mm) physiological foramina, respectively. The difference between the wide and narrow diameters in MaCas with one and two, three or four physiological foramina are within ranges of 0.02 to 0.08 and 0.06 to 0.11 mm, respectively. From a clinical point of view the wide diameter is the one that should be taken into consideration when determining the apical final preparation (MAF) size. Therefore, the wide diameter results and Weine’s^3^ recommendations were taken into consideration when evaluating the corresponding MAF suggestions and because the majority of physiological foramina observed were oval and these ideally should mechanically be prepared into a round shape. The MAFs recommended, according to the relative low differences between the wide and narrow diameters ranges computed in this research, are corresponding few. However, it should be mentioned that in the best case, with the tactile sense, an operator would be clinically able to detect the narrow and not the wide diameter, thus, making it difficult to give credit to and to follow the recommendations here given. Furthermore, the shape, cleanliness and hermetic sealing potential, not only of the physiological foramina but the entire root canal space, after the cleaning and shaping procedures is of importance. Concentrating the shaping procedures in one area could lead to structures weakening or even perforation^10,23,26^. Thus, a carefully considered and balanced decision when selecting the preparation method and system should be taken.

## Conclusions

- Single rooted mandibular canines (MaCas) were most frequently observed (97.0%) ones.
- The most frequently root canal configurations (RCC) observed in single-rooted MaCas were 1-1-1/1 (74.5%) and 1-2-1/2 (14.3%).
- Accessory and connecting canals were identified only in the middle and apical root thirds with a frequency up to 6.9%.
- Most MaCas had one physiological foramen (80.6%) with narrow and wide diameter mean sizes of 0.40 mm (± 0.11) and 0.28 mm (± 0.07), whereas most physiological foramina were oval shaped (91.1%).
- The potential for clinical relevance, when taking into consideration the results (specially the RCC and foramina shape) of this investigation, is that although an endodontic treatment of MaCas seems to be a straight forward one, ≈15% of treatment failures could be avoided when clinically being aware of, and able to recognize uncommon MaCas morphologies.
